# The Impact of Radiomics Image Analysis on Adult Hip Pathologies: A Scoping Review

**DOI:** 10.3390/jcm15041366

**Published:** 2026-02-09

**Authors:** Francesco Rosario Parisi, Biagio Zampogna, Alessandro Del Monaco, Giancarlo Giurazza, Emanuele Zappala, Andrea Zampoli, Augusto Ferrini, Domiziana Santucci, Elva Vergantino, Stefania Lamja, Eliodoro Faiella, Rocco Papalia

**Affiliations:** 1 Operative Unit of Orthopaedic and Trauma Surgery, Fondazione Policlinico Universitario Campus Bio-Medico, Via Alvaro del Portillo 200, 00128 Roma, Italy; f.parisi@policlinicocampus.it (F.R.P.); b.zampogna@policlinicocampus.it (B.Z.); g.giurazza@policlinicocampus.it (G.G.); emanuele.zappala@unicampus.it (E.Z.); andrea.zampoli@unicampus.it (A.Z.); augusto.ferrini@policlinicocampus.it (A.F.); r.papalia@policlinicocampus.it (R.P.); 2 Research Unit of Orthopaedic and Trauma Surgery, Department of Medicine and Surgery, Università Campus Bio-Medico di Roma, Via Alvaro del Portillo 21, 00128 Roma, Italy; 3 Department of Biomedical, Dental, Morphological and Functional Images (BIOMORF), University of Messina, A.O.U. Policlinico G. Martino, Via Consolare Valeria 1, 98124 Messina, Italy; 4 Operative Research Unit of Radiology and Interventional Radiology, Fondazione Policlinico Universitario Campus Bio-Medico, Via Alvaro del Portillo 200, 00128 Rome, Italy; d.santucci@policlinicocampus.it (D.S.); elva.vergantino@unicampus.it (E.V.); s.lamja@policlinicocampus.it (S.L.); e.faiella@policlinicocampus.it (E.F.); 5 Research Unit of Radiology and Interventional Radiology, Department of Medicine and Surgery, Università Campus Bio-Medico di Roma, Via Alvaro del Portillo 21, 00128 Rome, Italy

**Keywords:** radiomic, image analysis, hip pathologies

## Abstract

Radiomics promises quantitative biomarkers extracted from routine hip imaging to support diagnosis, prognosis, and surgical planning, but current evidence is fragmented across pathologies, modalities, and computational pipelines. We conducted a scoping review following PRISMA-ScR and the Population–Concept–Context framework, including peer-reviewed original studies on adults (≥18 years) that applied radiomics or deep-radiomics to hip imaging (X-ray, CT, MRI, DEXA) with clinically relevant outcomes. PubMed (MEDLINE), Embase and Scopus (Elsevier) were searched from 1 January 2021 to 30 August 2025 and complemented by snowballing; screening and data charting were performed in duplicate. Given heterogeneity, findings were synthesized narratively by a priori clusters. In fragility/osteoporosis, opportunistic CT and radiograph-based models frequently achieved AUCs around 0.90–0.96, while DXA-radiomics added information beyond bone mineral density/FRAX and trabecular MRI provided complementary microarchitectural signals. For osteonecrosis of the femoral head, multisequence MRI enabled early diagnosis with AUCs > 0.94; radiomics differentiated transient bone marrow edema with AUCs~0.92–0.94 and predicted collapse using radiographs or MRI with AUCs~0.85–0.90, including automated pipelines with external validation around 0.85. In femoroacetabular impingement, 3D Dixon-MRI studies reported very high performance (~0.97–1.00) with preliminary multicenter generalizability and added value from periarticular soft-tissue features. In total hip arthroplasty, radiomics anticipated press-fit cup stability from preoperative radiographs (AUC~0.82) and predicted 6-month functional recovery using clinico-radiomic CT models (AUC~0.95). Across clusters, methodological robustness was variable (sample sizes, harmonization, leakage control, external/temporal validation, calibration, clinical utility). Radiomics for adult hip disorders shows tangible translational promise in opportunistic screening, complex differential diagnosis, and perioperative decision support, but broader clinical adoption will require multicenter datasets, IBSI-aligned standardization, transparent reporting of calibration and decision-curve analyses, and prospective validation.

## 1. Introduction

Patients with hip osteoarthritis (OA) commonly exhibit similar radiographic findings, but they do not always present with the same clinical manifestations, with dramatically different pain and mobility. This clinical variability highlights the need for more precise tools to identify which patients with osteoporotic hips are at imminent risk of fracture and which patients with osteonecrosis of the femoral head are more likely to progress rapidly to collapse [1,2]. These queries illustrate a basic shortcoming of existing tools: their incapacity to completely capture the disease biology hidden behind the image. They also reflect everyday difficulties in orthopedic practice. This prognostic uncertainty is not an academic question but instead takes place within a massive epidemiological context. OA is a leading cause of global disability, with estimates exceeding 528 million people affected [3]. At the same time, healthcare systems are under strain due to the rise in femur fragility fractures, which have a one-year mortality rate of nearly 25% and cause survivors to drastically lose their independence [4,5]. Because of this, the overall number of total hip arthroplasty (THA) surgeries is continuously rising, with rates in OECD countries surpassing 170 procedures per 100,000 people [6]. Conditions such as femoro-acetabular impingement (FAI) in young adults and osteonecrosis of the femur (ONFH) also contribute to this clinical and economic burden [1,7]. The problem lies in the gap between what we see and what we can measure [2,8,9,10].

Conventional diagnostic tools, while indispensable, have well-known limitations. The fundamental tools for determining fracture risk are bone densitometry (DXA) and scores like FRAX, but they frequently fall short in describing the microarchitecture and inherent quality of bone [8,9,11,12]. In a similar vein, methodological variations and disputed thresholds affect the morphometric indices used for FAI [7,13]. At the same time, the early diagnosis of ONFH on magnetic resonance imaging (MRI), based on qualitative interpretation, can be influenced by interobserver variability [14]. To bridge this gap, radiomics and deep radiomics emerged, a field that treats medical images not as simple photographs, but as dense matrices of data [15]. Radiomics uses computer algorithms to extract hundreds of quantitative features from standard tests like MRIs, CT scans, and X-rays [15,16]. By describing the tissue’s shape, intensity, and most importantly, texture, these features function as a kind of digital microscope that makes visible features that are not visible to the naked eye [15]. Here, radiomics refers to the extraction of predefined (‘hand-crafted’) quantitative features from medical images, whereas deep radiomics refers to imaging features automatically learned using deep learning (typically CNN-based representations), either alone or combined with hand-crafted features in hybrid models. The objective is to turn this obscure data into reliable biomarkers that can be utilized to create predictive models that can assist with well-informed clinical judgments [16,17]. The field of applying radiomics to hip pathologies is currently rich but fragmented, despite its potential [2,16,17,18,19]. The existing literature is heterogeneous in terms of the pathology studied, imaging modality, computational pipeline, and level of validation [16,17,18,19]. This diversity makes it challenging for clinicians and researchers to gain a clear understanding, identify best practices, and recognize the real gaps that need to be addressed. Because of this complexity, we chose a scoping review to map a rapidly evolving and heterogeneous field across pathologies, imaging modalities and computational pipelines where a formal effect size synthesis is premature and the primary aim is to identify concepts, methods, and gaps rather than to pool estimates. We conducted a scoping review in a methodical approach in order to: (i) identify clinical applications and outcomes that have already been studied; (ii) outline current methodological trends; and (iii) highlight important gaps and suggest solutions. Ultimately, this work aims to provide a solid foundation for guiding future research and accelerating the translation of radiomics into clinical practice.

## 2. Materials and Methods

### 2.1. Design and Protocol

This scoping review was conducted following the methodological and reporting guidelines defined by PRISMA Extension for Scoping Reviews (PRISMA-ScR) [20]. A prospective registration was not performed. We followed the Population–Concept–Context (PCC) framework: the Population of Interest was defined as adult patients aged 18 years and older with hip disease, the central concept concerned the application of radiomics or deep radiomics approaches to hip imaging and the context included any clinical or research setting that employed imaging modalities such as conventional radiography (XR), computed tomography (CT), magnetic resonance imaging (MRI), or dual-energy X-ray absorptiometry (DXA). Conventional radiomics was defined as the extraction of pre-defined (‘hand-crafted’) quantitative features. Deep radiomics was defined as the use of deep learning–derived imaging representations (e.g., CNN-extracted features/embeddings) as quantitative descriptors for predictive modeling, either alone or in hybrid clinico-imaging models; such approaches were considered within our inclusion concept.

### 2.2. Eligibility Criteria

Eligible studies were original studies published in peer-reviewed journals involving an adult population and applying a radiomics pipeline to a hip adult pathologies. Furthermore, studies that, despite not having direct clinical application, described and validated key methodological steps for hip radiomics, such as the development of automatic segmentation algorithms, were also included. Case reports, case series, studies on pediatric populations, literature reviews, editorials, conference abstracts, and any preclinical studies conducted on animals, cadavers, or phantoms, as well as studies not relevant to the hip, were excluded. Studies using CNN-based feature extraction were eligible when the learned imaging features were used as quantitative inputs for classification or prediction (deep radiomics), alone or combined with hand-crafted radiomic features.

### 2.3. Sources of Information and Search Strategy

The literature search was conducted on the electronic databases PubMed (MEDLINE), Embase and Scopus (Elsevier). The search period was set from 1 January 2021, to 30 August 2025. This restricted time frame was defined to only consider studies with methodological parameters that reflect the state of the art in the field and to capture pipelines aligned with IBSI-compliant feature extraction, modern harmonization, and contemporary ML validation practices; earlier works are not charted. The search strategy performed an analysis using free-text searches, entered in the title and abstract fields, as well as indexed terms such as Medical Subject Headings (MeSH). Additional details are provided in the Supplementary Materials (File S1).

### 2.4. Selection Process

**Figure 1:**
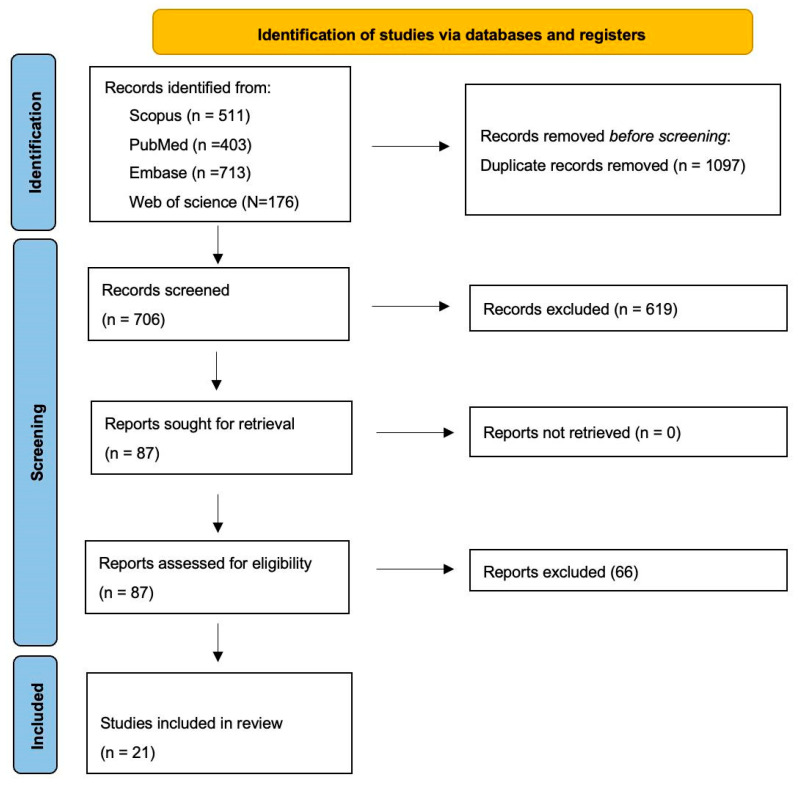
(PRISMA) flowchart.

After importing all records into Rayyan^®^ (Rayyan Systems Inc., Cambridge, MA, USA) to remove duplicates, two reviewers independently screened titles and abstracts. Prior to formal screening, a calibration exercise was performed on 100 records to harmonize eligibility interpretation. The same reviewers then independently assessed the full texts of potentially eligible articles. Any disagreements were resolved by consensus; if consensus was not reached, a third reviewer adjudicated. The study selection process, from identified records to the final included studies, is reported using a PRISMA flow diagram (Figure 1).

### 2.5. Data Extraction (Data Charting)

Data extraction was performed in duplicate by two independent reviewers using a predefined and standardized form, implemented on a shared spreadsheet Google Sheets, (Google LLC, Mountain View, CA, USA) to facilitate collaboration. The form was designed to systematically collect general information such as authors, year, study design, and sample size. Clinical and imaging data were extracted, including the pathology investigated and the modality used, along with comprehensive details of the radiomics pipeline. This final section included the segmentation method with the related accuracy and reproducibility metrics; the characteristics of feature extraction and selection, such as the software, feature families, and selection algorithms; and modeling details, such as the machine learning algorithm, the use of harmonization techniques, the use of interpretability tools and the validation framework adopted. Finally, all parameters related to function and performance, including AUC (area under the curve), accuracy, sensitivity and specificity, were collected and further complemented by calibration and clinical utility analyses, such as the study of decision curves. For deep radiomics studies, we recorded the use of CNN-derived imaging features/embeddings and whether they were used alone or within hybrid models with hand-crafted radiomic features.

### 2.6. Methodological Appraisal

In line with the objectives of a scoping review, which aims to map the extent and nature of the evidence rather than formally assess its quality, a critical risk-of-bias assessment of individual studies was not performed. Nonetheless, to contextualize the results and guide their interpretation, several key indicators of methodological robustness were recorded. They will be discussed in the narrative synthesis, including the presence of external or multicenter validation, the adoption of strategies to control overfitting, and reporting on process reproducibility.

### 2.7. Data Synthesis

Considering the expected high heterogeneity among studies in terms of pathologies, imaging modalities, computational pipelines, and outcomes, a quantitative meta-analysis was not planned. The synthesis of results will therefore be narrative and structured into predefined thematic clusters based on the pathology (e.g., Osteoporosis and Frailty; ONFH; FAI; THA). Within each cluster, common characteristics will be described, reported performance will be discussed, and recurring limitations and knowledge gaps will be highlighted. Detailed comparative tables and graphical visualizations will support the presentation of the distribution of evidence.

### 2.8. Performance Extraction and Hierarchy

To minimize optimism bias and enhance clinical interpretability, we pre-specified an extraction hierarchy. For each study we recorded the primary endpoint and reported the discrimination metric (AUC or c-index) from the highest validation level available in the following order: external independent test, temporal validation, internal hold-out test, nested cross-validation (reported as mean ± SD or median [IQR]). Apparent (training) performance was not extracted. Where multiple models/pipelines were presented, we selected the model with the highest validation level; ties were resolved in favor of models reporting calibration and/or decision-curve analysis and with fewer predictors. Additional metrics (sensitivity/specificity/PPV/NPV, calibration, decision-curve) are reported only when derived from the same dataset as the primary discrimination metric; for PPV/NPV we recorded the prevalence of the test set when available. For time-to-event outcomes we prioritized the c-index; if unavailable, we used AUC at a pre-specified horizon consistent across studies whenever possible. For multi-class tasks we reported macro-averaged discrimination unless otherwise stated.

## 3. Results

### 3.1. Fragility and Osteoporosis

This thematic cluster groups together studies focused on identifying osteoporosis and estimating fracture risk (Table 1). The emerging evidence demonstrates that radiomics, applied to different imaging modalities, offers significant added value compared to current standards [2,21,22,23]. In particular, pipelines based on CT and X-ray showed excellent diagnostic performance, with AUC frequently ranging between 0.90 and 0.96 [21,24,25,26]. In parallel, radiomics applied to DXA has been shown to enhance risk stratification beyond conventional parameters, such as T-scores and FRAX [2]. At the same time, trabecular MRI analysis provides complementary microarchitectural information [23]. The most promising application is opportunistic screening via CT. Several studies have demonstrated that abdominal-pelvic (APCT) or low-dose (LDCT) scans, performed for other indications, can classify bone fragility status with high accuracy [24,25,27]. Integrated analysis of radiomic features with mean measurements in Hounsfield Units (HU) showed superior results compared to individual approaches in predicting individual fracture risk [22]. This evidence is extremely promising for the clinical translation of these algorithms, which eliminate the need for manual intervention, making these pipelines scalable and efficient [21,25,27]. This is confirmed by time-based validations that demonstrate their robustness (25). These models have achieved very high specificity and negative predictive values (NPV) (~95–98%), making them ideal tools for definitively ruling out osteoporosis and referring only equivocal cases to DXA, thereby optimizing the process [24,25]. Traditional imaging modalities also benefit significantly from this approach. On conventional XR, CNN-derived imaging feature models (deep radiomics), combined with clinical data have achieved AUCs of up to 0.95 in external validation cohorts, exceeding the average performance of radiologists and significantly improving it when used as a “second reader” system [21]. On DXA, the extraction of texture features has allowed the creation of radiomics scores capable of predicting fracture risk independently and additively with respect to T-scores and FRAX, capturing aspects of bone quality not reflected by mineral density alone [2]. Finally, radiomic analysis of trabecular MRI was shown to be able to discriminate between patients with and without fragility fractures. Although with more modest performance (AUC~0.72–0.75), the extracted features showed a weak correlation with DXA parameters, suggesting that MRI captures unique microstructural information [23]. This positions it as a complementary tool, helpful in enriching the risk profile in patients undergoing hip MRI for other reasons.

### 3.2. Femoroacetabular Impingement (FAI)

Studies involving patients with FAI present a surprisingly consistent and promising picture (Table 2). Several trials have demonstrated that radiomics, applied to Dixon’s 3D magnetic MRI sequences, can classify FAI-affected hips with high accuracy, distinguishing them from healthy hips [19,28]. This approach has been shown to outperform traditional radiological measurements and, crucially for clinical translation, to maintain high performance even in multicenter validation settings, suggesting good generalizability of the radiomics signal [28,29]. Initial studies have established that 3D Dixon MRI images contain a strong and discriminatory radiomics signal. Machine learning models, based on a limited number of features, have achieved accuracies and AUCs close to 0.97 in differentiating FAI-affected hips from healthy hips in the same patient [19,28]. It emerged that, in addition to three-dimensional shape features, texture features (e.g., GLCM, GLRLM) and gray-level distribution (first-order) were also highly informative. The latter captures the heterogeneity of bone microstructure, a possible reflection of mechanical stress and remodeling induced by the conflict [19,28]. A comparison confirmed the superiority of radiomics, which achieved a classification accuracy of 100% compared to the ~74% achieved with a set of 21 standard radiological measurements, highlighting how multiparametric analysis is able to capture variations not detected by individual morphometric indices [28]. The robustness of these models was confirmed by a multicenter external validation study. A model trained on data from a single center correctly identified cases of symptomatic FAI provided by other centers, which used different acquisition protocols. Interestingly, in this study, features extracted from the gluteal muscles were also found to be informative, suggesting that analyzing the surrounding soft tissue adds valuable context to the classification [29]. Finally, on the methodological front, automatic 3D segmentation of the femur and acetabulum using neural networks (3D U-Net) is achievable. One study demonstrated that a targeted data augmentation strategy is more effective than transfer learning from other anatomical regions for obtaining accurate segmentation masks (Dice score > 0.84), a prerequisite for automating the entire radiomics pipeline [30].

### 3.3. Osteonecrosis of the Femoral Head (ONFH)

For ONFH, the radiomics literature primarily focuses on two crucial clinical challenges: early and differential diagnosis, as well as prediction of femoral head collapse (Table 3). The developed models consistently demonstrate robust performance, with AUC values ranging between 0.85 and 0.95 [18,31,32,33,34]. A recurring and impactful theme is that these tools match or surpass the performance of less experienced clinicians in diagnostic and prognostic tasks, suggesting strong potential for standardizing and improving clinical accuracy [18,31,32,34]. In the context of MRI diagnosis, radiomics has proven particularly effective. Models based on multi-sequence images (T1, T2, STIR) achieved AUCs greater than 0.94 in recognizing early ONFH, demonstrating accuracy superior to that of radiology residents [18]. The approach has also proven powerful in resolving complex differential diagnoses. For example, SHAP-interpretable models distinguished ONFH from osteoarthritis (AUC ≈ 0.97) more accurately than orthopedists and radiologists, while also providing an explanation for the most influential features (e.g., head sphericity, texture heterogeneity) [31]. Similarly, radiomics has been shown to differentiate ONFH from transient bone marrow edema (AUC ≈ 0.92), a crucial task for avoiding inappropriate treatments for a self-resolving condition [32,33]. In predicting femoral head collapse, radiomics has been shown to extract valuable prognostic information from different imaging modalities. Models based on plain XR predicted collapse at two years with an AUC of approximately 0.90, surpassing the predictive capacity of orthopedists and demonstrating that even low-cost tests contain quantitative patterns of structural fragility [34]. To make this approach scalable, fully automated pipelines on MRI were developed that, using neural networks (nnU-Net) for segmentation, predict collapse with good accuracy (AUC ≈ 0.85) in external validation settings, a key step for integration into the clinical workflow [35]. A clinico-radiomic nomogram, combining CT-extracted features with lipid biomarkers (triglycerides, HDL), has been shown to be effective in predicting the risk of steroid-induced ONFH. The credibility of this result is strengthened by transcriptomic analyses that confirm the potential of hybrid models to provide more comprehensive and personalized risk stratification [36].

### 3.4. TOH (Transient Bone Marrow Edema) vs. ONFH

Distinguishing between transient edema of the hip (TOH/TBMES) and ONFH is a significant challenge (Table 4). Studies in this cluster demonstrate how radiomics, applied to a routine MRI sequence, provides a powerful quantitative tool to resolve this ambiguity, with performance that rivals that of subspecialist radiologists [32,33]. Radiomics analysis of standard, noncontrast sequences has proven highly effective. An STIR-based model with an AUC of 0.937 was able to differentiate TOH from ONFH in a large-scale study. The model’s performance was noticeably better than that of a general radiologist and on par with two radiologists with musculoskeletal pathology (MSK) experience [32]. The most informative features were found to be almost exclusively wavelet-type indices that capture the heterogeneity of the subchondral bone texture at different scales. This suggests that the model is not based on simple differences in intensity, but "sees" complex microstructural patterns that reflect the different pathophysiology of diffuse edema (in TOH) versus focal necrosis (in ONFH) [32]. Another study confirmed these results using the T1 sequence and a pragmatic design based on clinical follow-up to establish the ground truth diagnosis. An SVM model predicted with an AUC of 0.92 which edematous lesions would resolve spontaneously and which would progress to full-blown necrosis [33]. With this method, follow-up and treatment decisions can be guided by an earlier prognosis based on the initial scan. To sum up, radiomics on standard MRI sequences (STIR, T1) offers a reliable, approachable, and expandable solution to this important diagnostic conundrum. It does not call for lengthy protocols or the use of contrast media, in contrast to more sophisticated techniques like DCE-MRI. By providing a quantitative evaluation consistent with the specialist’s field of expertise, it can serve as a support system, lowering the likelihood of management mistakes and interpretive variability in routine clinical practice [32,33].

### 3.5. THA: Preoperative Planning and Functional Prognosis

In the context of THA, radiomics is a valuable decision support tool throughout the perioperative care process (Table 5). The emerging studies focus on two high-value clinical applications: preoperative planning, through the estimation of implant stability, and postoperative prognosis, through the prediction of functional recovery. Both applications utilize data and images already collected in routine practice, providing solutions with minimal impact on clinical workflow [37,38]. For preoperative planning, a study demonstrated the possibility of predicting the primary stability of a cementless (press-fit) acetabular cup based on a simple pelvic X-ray. Using the intraoperative stability test as a reference, a machine learning model (XGBoost) achieved an AUC of 0.82. This result is clinically relevant because it transforms an assessment previously based on the surgeon’s intraoperative experience into a quantitative preoperative indication. This information can guide crucial surgical decisions in advance, such as the degree of reaming (under-reaming) or the need for accessory fixation, to reduce the risk of early implant loosening. Radiomics, in this case, translates the texture patterns of the acetabular bone visible on the X-ray into a usable risk estimate [37]. For postoperative prognosis, another study focused on predicting functional recovery (Harris Hip Score ≥ 90) six months after THA for femoral neck fracture. The best-performing approach was a clinical-radiomic model that integrated features extracted from preoperative CT with patient demographics and hemostatic parameters (e.g., APTT, fibrinogen). With an outstanding AUC of 0.95, this hybrid model outperformed models that were only based on clinical or radiomic data. This outcome emphasizes the benefits of integrating the patient’s systemic biological status with the local bone imaging phenotype. Such a prognostic tool can help calibrate patient expectations and personalize rehabilitation pathways, enhancing support for patients with a lower probability of optimal recovery [38].

## 4. Discussion

This scoping review has mapped a rapidly expanding field of research, demonstrating how radiomics has established itself as a mature paradigm applicable to the entire spectrum of adult hip pathologies. The evidence collected outlines pre-clinical translation trajectories in critical areas like surgical planning, differential diagnosis, and frailty screening, going beyond the simple proof-of-concept stage. Beyond the methodological heterogeneity, some noteworthy convergent trends show up, such as the move toward automation for segmentation through deep learning, the enhancement of clinical-radiomic model performance, the first simple examples of external validation, and an increasing focus on model interpretability. Despite these advances, significant gaps remain that future research must address to enable widespread adoption.

First, a powerful message emerging from this mapping is the advent of opportunistic intelligence for bone health. Radiomics enables the extraction of quantitative biomarkers from tests, such as abdominal-pelvic CT scans or pelvic radiographs, performed for other indications. This "low-friction" method turns ordinary data into effective osteoporosis screening tools with outstanding diagnostic performance (AUCs ranging from 0.90 to 0.96), which is occasionally validated by external or temporal validation [21,24,25]. The near-perfect automation of the pipelines [25,27] and their efficacy even at low doses underscore their feasibility on a large scale. At the same time, radiomics also extracts added value from dedicated tests, such as DXA, providing prognostic scores for fracture risk that are proven to be independent and complementary to conventional parameters, including BMD and FRAX [2]. These findings collectively support a new triage model in which radiomics serves as an intelligent filter to identify patients at risk for referral to specific prevention pathways.

The second cross-cutting theme is the role of radiomics as a decision support tool in complex diagnostic dilemmas. In settings where qualitative assessment may be uncertain or dependent on the observer’s experience, radiomics offers a quantitative and reproducible “second opinion.” In the differential diagnosis between TOH and ONFH a critical clinical crossroads radiomic models achieved performance (AUC~0.92–0.94) comparable to that of subspecialist radiologists (MSK) and superior to that of generalists [32,33]. Similarly, the models performed better than residents in external validation for the early detection of ONFH [18]. In addition to achieving near-perfect accuracies for femoroacetabular impingement (FAI) using radiomics on Dixon 3D MRI [19,28,29], a multicenter validation study showed strong generalizability [29,31]. Several applications in this field report exceptionally high or near-perfect descriptive and discriminative performance (e.g., in FAI classification and selected predictive contexts). While encouraging, these results require caution, as they may partly reflect optimism bias rather than true generalization ability. In radiomics, very high AUCs can be exaggerated by small sample sizes or homogeneous single-center cohorts. Furthermore, subtle forms of data loss (preprocessing or feature selection performed before splitting between training and testing) can artificially inflate performance estimates. Therefore, extreme metrics should be considered hypothesis-generating unless supported by independent external validation and transparent reporting of the entire pipeline. Ultimately, radiomics is making a strong entry into the field of prosthetic surgery by helping patients during the perioperative period. Despite being preliminary, the evidence is encouraging. A model based on standard radiographs could be used to predict the press-fit stability of the acetabular implant preoperatively (AUC~0.82), providing surgeons with quantitative information to customize their surgical strategy [37]. Postoperatively, a hybrid model demonstrated the power of synergy between local phenotype and systemic biology, combining radiomic features from preoperative CT with hemostatic biomarkers to predict functional recovery at six months with excellent accuracy (AUC~0.95), outperforming models based solely on clinical or imaging findings [38]. This approach paves the way for a personalized prognosis, allowing expectations to be calibrated and rehabilitation pathways to be tailored. Despite these encouraging results, translation into clinical practice requires addressing the methodological gaps that have emerged. The diversity of pipelines and the absence of standardization in feature selection and preprocessing continue to be the primary obstacles. External validations are not yet a standard practice, despite the fact that they are starting to appear [18,21,29,35,36]. Importantly, the rapid methodological maturation of the field should not be conflated with clinical readiness. Across adult hip disorders, most evidence remains pre-implementation: studies are predominantly retrospective, validation is often internal, and reporting of robustness elements is inconsistent. Accordingly, this scoping review is intended to map feasible use-cases and emerging trajectories rather than to certify deployability. Progress toward routine adoption will require multi-center external validation on heterogeneous datasets, prospective evaluation in clinically representative cohorts, and demonstration of incremental value over established clinical predictors within decision-making workflows.

Future research should primarily focus on the creation of public, multicenter datasets to test the generalizability of models and promote the development of shared standards. The integration of radiomics with other omics disciplines (genomics, transcriptomics), as suggested by Jia [36] represents a further, fascinating frontier for enriching patient profiling and understanding underlying biological mechanisms.

## 5. Conclusions

Radiomics applied to adult hip disorders has moved beyond the exploratory phase, consolidating itself as a discipline with concrete and diverse translational potential. This scoping review has mapped a growing body of evidence supporting the use of radiomics along three main clinical lines: low-cost, opportunistic screening for bone fragility, support for differential diagnosis in complex conditions such as osteonecrosis, and guidance for planning and prognosis in prosthetic surgery. Quantitative models have demonstrated the ability to extract biologically plausible and clinically relevant information from routine examinations, often achieving performance comparable or superior to that of non-expert readers.

However, the transition from research to daily clinical practice is not automatic. The scientific community’s ability to overcome present obstacles, build and implement sizable multicenter databases, and conduct prospective studies will be crucial to turning the promising results of radiomics into advantages for patients and advancing an even more accurate and successful approach to the treatment of hip disorders.

## Figures and Tables

**Table 1:** Fragility and Osteoporosis radiomics across imaging modalities: summary of studies evaluating radiomics for osteoporosis detection and fragility-fracture risk stratification across Computed Tomography (opportunistic APCT/LDCT), XR, DXA and trabecular MRI. APCT = abdominal–pelvic Computed Tomography; LDCT = low-dose Computed Tomography; XR = radiograph; DXA = dual-energy X-ray absorptiometry; QCT = quantitative Computed Tomography; BMD = bone mineral density; FRAX = Fracture Risk Assessment Tool; HU = Hounsfield Units; AUC = area under the ROC curve; ROI = region of interest.

Study	Condition	Modality/Roi	Segmentation	Model	Validation	Performance	Outcome
Hong et al., 2020 [2]	Hip fracture risk	DXA hip (FN, TR, IT, TH)	Manual in 3D Slicer (ICC ≥ 0.90 in 78%)	Random Forest (Elastic Net/GBM/SVM also tested)	Hold-out + prospective cohort	External test AUC 0.705; HR 1.04–1.06/unit	Fracture prediction, incremental to BMD & FRAX
Yuan et al., 2025 [22]	Hip fracture risk	CT hip/pelvis; proximal femur VOI	Semi-auto (TotalSegmentator + SAM + ITK-SNAP); ICC > 0.90	Logistic Regression; Combined LR + HU	Internal + two independent tests	External test Radiomics AUC 0.875–0.798; Combined AUC 0.934–0.893–0.851	Identify high-risk for fragility fracture
Martel et al., 2023 [23]	Hip fracture risk	3T MRI FLASH T1; trabecular bone	Manual (FireVoxel)	Univariate ROC (no ML)	Internal only	Internal hold-out Radiomics AUROC 0.72–0.75; DXA~0.52–0.59	Fracture vs. non-fracture discrimination
Du et al., 2025 [27]	Bone status	LD abdominal CT; proximal femur	VB-Net auto-seg (DSC 0.975/0.955)	Random Forest (3-class)	Train/val/test; QCT as reference	Internal hold-out AUC: N 0.924; OPn 0.828; OP 0.960	Opportunistic triage of bone status
Park et al., 2024 [25]	Osteoporosis vs. non osteoporosis	APCT pre-contrast; left proximal femur	DL autoseg (technical success 99.7%)	Random Forest (undersampling; 5-fold CV)	Temporal validation	Temporal Validation AUC 0.946; Spec 98.1%	Opportunistic osteoporosis screening
Lim et al., 2021 [24]	Femoral osteoporosis	APCT pre-contrast; left proximal femur	Semi-auto region growing (ICC ≥ 0.90 kept)	Random Forest (random search; 5-fold CV)	Train/val 70/30 split	Internal hold-out AUC 0.959/0.960; Acc ~92.7%; Spec ~95.8%	Opportunistic OP screening on APCT
Kim et al., 2022 [21]	Osteoporosis	Hip AP radiographs; auto-seg	Fusion-Net U-Net (Dice 0.98)	MLP on deep + texture + clinics (DTC)	Internal + external + observer study	External test AUC 0.95; >radiologists;	XR-based opportunistic OP screening
Fang et al., 2024 [26]	Osteoporosis	Hip CT; whole hip ROI	Semi-auto (ITK-SNAP)	GradientBoosting (rad-only); XGBoost (fusion)	Train/test split	Internal hold-out AUC 0.919; Fusion AUC 0.886	Classify OP vs. non-OP

**Table 2:** Femoroacetabular impingement (FAI) radiomics on 3D Dixon MRI: Synthesis of studies applying radiomics to 3D Dixon hip MRI for FAI detection and phenotyping. FAI = femoroacetabular impingement; MRI = magnetic resonance imaging; ROI = region of interest; AUC = area under the ROC curve; Acc = accuracy; CV = cross-validation; DSC = Dice similarity coefficient; GLCM = gray-level co-occurrence matrix; GLRLM = gray-level run-length matrix; k-NN = k-nearest neighbors; RF = random forest; LGBM = Light Gradient Boosting Machine; STAPLE = simultaneous truth and performance level estimation.

Study	Condition	Modality/Roi	Segmentation	Model	Validation	Performance	Outcome
Montin et al., 2024 [28]	FAI diagnosis	3T MRI Dixon 3D; femur + acetabulum	Semi-auto ITK-SNAP; radiologist QA	Random Forest; 100 × 5-fold CV	Internal resampling; no external	Cross Validation: AUC 1.00 (ALL subset); ~0.99 on acetabular FO/IN	Classify FAI vs. healthy
Montin et al., 2025 [29]	FAI symptomatic vs. asymptomatic vs. healthy	3D Dixon MRI; bone + soft tissues	Automatic (TotalSegmentator + STAPLE)	LGBM/RFC/Bagging best	Internal 4-fold; External multicenter	External test: 100% symptomatic detection (several models)	Generalizable classification across sites/scanners
Montin et al., 2024 [30]	DL segmentation performance	3D Dixon MRI; femur + acetabulum	3D U-Net; DA vs. TL	U-Net variants	Hold-out test; comparative configs	Internal hold-out Dice up to 0.893 (femur), 0.842 (acetabulum)	Segmentation accuracy for radiomics pipeline
Montin et al., 2023 [19]	FAI diagnosis	3D Dixon water-only MRI; femur + acetabulum	Manual (ITK-SNAP)	k-NN (k = 3)	100× CV + hold-out	Internal hold-out AUC/Acc~0.97	Distinguish FAI vs. healthy

**Table 3:** Osteonecrosis of the Femoral Head (ONFH) radiomics for early/differential diagnosis and collapse prediction. Summary of studies evaluating radiomics across MRI (T1, T2-FS, STIR), plain radiographs, and CT to (i) detect early ONFH and differentiate it from OA and TOH/TBMES, and (ii) predict femoral head collapse. ONFH = osteonecrosis of the femoral head; TOH/TBMES = transient osteoporosis of the hip / transient bone marrow edema syndrome; FS = fat-suppressed; LR = logistic regression; SVM = support vector machine; XGB = extreme gradient boosting; SHAP = SHapley Additive exPlanations;.

Study	Condition	Modality/Roi	Segmentation	Model	Validation	Performance	Outcome
Batur et al., 2023 [33]	TBMES vs. ONFH	MRI 3T T1; 2D ROI	Manual (IBEX); ICC intra 0.84–0.87; inter 0.86–0.89	SVM best; RF as comparator	Internal split 70/30 + 1000 bootstrap	External test: SVM AUC 0.921; Sens 91.3%; Spec 85.1%	Differentiation reversible vs. irreversible BMLs
He et al., 2025 [34]	ONFH collapse prediction (2 years)	X-ray AP + FL; necrosis ROI	Manual (ITK-SNAP); MRI-assisted referencing	SVM best (RF/SGD tested)	External test set + internal CV	External test: AUC 0.904; Sens 81.8%; Spec 79.5%; >surgeons (AUC 0.50–0.65)	Predict collapse risk to guide management
Wang et al., 2024 [18]	Early ONFH diagnosis	MRI T1/FS-T2/STIR; VOI head → near lesser trochanter	3D Slicer; ICC checked; ComBat harmonization	LASSO-LR (single/dual/multi-seq)	Train/val (Inst A) + external test (Inst B)	External test: AUC 0.961/0.957/0.938; Acc test 87.5% (beats residents)	Objective early ONFH diagnosis
Jia et al., 2024 [36]	Steroid-induced ONFH risk	CT proximal femur	Semi-auto (3D Slicer)	Clinical (TG, HDL) + Rad-score nomogram	Internal + external multicenter	External test: AUC 0.991/0.915/0.901	Risk prediction of SONFH onset
Gao et al., 2024 [35]	ONFH collapse prediction	MRI T1; nnU-Net auto-seg of necrotic lesion	nnU-Net (DSC 0.848)	LightGBM best (LR/RF/SVM/KNN/XGB)	External test (Center 2)	External test: AUC 0.851;	Predict collapse within 2 years
Alkhatatbeh et al., 2025 [31]	ONFH vs. OA	MRI T2 FS; head+neck/necrotic area	Manual (ITK-SNAP)	Naive Bayes (SHAP interpretability)	70/30 split	Internal hold-out: AUC 0.971; Acc 90.5%; Sens 93.7%; Spec 88.5%	Differential diagnosis

**Table 4:** Transient Bone Marrow Edema (TOH/TBMES) vs. Osteonecrosis of the Femoral Head (ONFH) radiomics on standard hip MRI. Summary of studies differentiating TOH/TBMES from ONFH using routine, non-contrast MRI sequences.

Study	Condition	Modality/Roi	Segmentation	Model	Validation	Performance	Outcome
Batur et al., 2023 [33]	TBMES vs. ONFH	MRI 3T T1; 2D ROI	Manual (IBEX); ICC intra 0.84–0.87; inter 0.86–0.89	SVM best; RF as comparator	Internal split 70/30 + 1000 bootstrap	Internal hold-out: SVM AUC 0.921; Sens 91.3%; Spec 85.1%	Differentiation reversible vs. irreversible BMLs
Klontzas et al., 2021 [32]	TOH vs. AVN	MRI STIR; proximal femur	Manual (3D Slicer)	XGBoost best (CatBoost/SVM also)	70/30 split; multivendor	Internal hold-out: AUC 0.937; Sens 93.6%; Spec 93.9%	Differential diagnosis

**Table 5:** Total hip arthroplasty (THA) radiomics for preoperative planning and functional prognosis: Summary of studies leveraging routine imaging to (i) anticipate primary press-fit cup stability from preoperative pelvic radiographs and (ii) predict functional recovery 6 months after THA using clinico-radiomic models from preoperative CT. THA = total hip arthroplasty; HHS = Harris Hip Score; APTT = activated partial thromboplastin time.

Study	Condition	Modality/Roi	Segmentation	Model	Validation	Performance	Outcome
He B. et al., 2024 [37]	Acetabular press-fit stability (THA)	AP pelvic XR; acetabular bone ROI	Manual (3D Slicer)	XGBoost best	Internal split 80/20; 5-fold CV	Internal hold-out: AUC test 0.823; Acc 78.9%; Sens 83.3%; Spec 75.0%	Predict stable vs. unstable press-fit
Zheng et al., 2022 [38]	Prognosis (HHS ≥ 90 at 6 months)	CT hip; pre and post-op	Not specified (PyRadiomics-based masks)	Random Forest (clinico ± radiomics)	Random split 7:3	Internal hold-out: (preop clinico+radiomics) AUC 0.949 (test)	Predict functional recovery after THA

## Data Availability

No new data were created or analyzed in this study.

## Supplementary Materials

The following supporting information can be downloaded at: https://www.mdpi.com/article/10.3390/jcm15041366/s1, File S1—Full Search Strategy (Databases, Strings, Filters, and Deduplication).

## References

[1] Mont M.A., Salem H.S., Piuzzi N.S., Goodman S.B., Jones L.C. (2020). Nontraumatic Osteonecrosis of the Femoral Head: Where Do We Stand Today?: A 5-Year Update. J. Bone Jt. Surg..

[2] Hong N., Park H., Kim C.O., Kim H.C., Choi J.-Y., Kim H., Rhee Y. (2020). Bone Radiomics Score Derived From DXA Hip Images Enhances Hip Fracture Prediction in Older Women. J. Bone Miner. Res..

[3] Steinmetz J.D., Culbreth G.T., Haile L.M., Rafferty Q., Lo J., Fukutaki K.G., Cruz J.A., Smith A.E., Vollset S.E., Brooks P.M. (2023). Global, regional, and national burden of osteoarthritis, 1990–2020 and projections to 2050: A systematic analysis for the Global Burden of Disease Study 2021. Lancet Rheumatol..

[4] Hertz K., Santy-Tomlinson J. (2018). Fragility Fracture Nursing: Holistic Care and Management of the Orthogeriatric Patient; Perspectives in Nursing Management and Care for Older Adults.

[5] Buecking B., Timmesfeld N., Riem S., Bliemel C., Hartwig E., Friess T., Liener U., Ruchholtz S., Eschbach D. (2013). Early Orthogeriatric Treatment of Trauma in the Elderly. Dtsch Ärztebl Int..

[6] Jennison T., MacGregor A., Goldberg A. (2023). Hip arthroplasty practice across the Organisation for Economic Co-operation and Development (OECD) over the last decade. Ann. R. Coll. Surg. Engl..

[7] Frank J.M., Harris J.D., Erickson B.J., Slikker W., Bush-Joseph C.A., Salata M.J., Nho S.J. (2015). Prevalence of Femoroacetabular Impingement Imaging Findings in Asymptomatic Volunteers: A Systematic Review. Arthrosc. J. Arthrosc. Relat. Surg..

[8] Silva B.C., Broy S.B., Boutroy S., Schousboe J.T., Shepherd J.A., Leslie W.D. (2015). Fracture Risk Prediction by Non-BMD DXA Measures: The 2015 ISCD Official Positions Part 2: Trabecular Bone Score. J. Clin. Densitom..

[9] Martínez-Montoro J.I., García-Fontana B., García-Fontana C., Muñoz-Torres M. (2022). Evaluation of Quality and Bone Microstructure Alterations in Patients with Type 2 Diabetes: A Narrative Review. J. Clin. Med..

[10] Turmezei T.D., Treece G.M., Gee A.H., Sigurdsson S., Jonsson H., Aspelund T., Gudnason V., Poole K.E.S. (2020). Quantitative 3D imaging parameters improve prediction of hip osteoarthritis outcome. Sci. Rep..

[11] Initiative T.F.O.T.F., Kanis J.A., Hans D., Cooper C., Baim S., Bilezikian J.P., Binkley N., Cauley J.A., Compston J.E., Dawson-Hughes B. (2011). Interpretation and use of FRAX in clinical practice. Osteoporos. Int..

[12] Goel H., Binkley N., Boggild M., Chan W.P., Leslie W.D., McCloskey E., Morgan S.L., Silva B.C., Cheung A.M. (2024). Clinical Use of Trabecular Bone Score: The 2023 ISCD Official Positions. J. Clin. Densitom..

[13] Martin R.K., Dzaja I., Kay J., Memon M., Duong A., Simunovic N., Ayeni O.R. (2016). Radiographic outcomes following femoroacetabular impingement correction with open surgical management: A systematic review. Curr. Rev. Musculoskelet. Med..

[14] Schmitt-Sody M., Kirchhoff C., Mayer W., Goebel M., Jansson V. (2008). Avascular necrosis of the femoral head: Inter- and intraobserver variations of Ficat and ARCO classifications. Int. Orthop..

[15] Gillies R.J., Kinahan P.E., Hricak H. (2016). Radiomics: Images Are More than Pictures, They Are Data. Radiology.

[16] Oh J., Hong S.H., Choi J.Y., Yoo H.J., Chae H.D. (2025). Radiomics in non-oncologic musculoskeletal diseases: From pixels to practice. Clin. Radiol..

[17] Fritz B., Yi P.H., Kijowski R., Fritz J. (2023). Radiomics and Deep Learning for Disease Detection in Musculoskeletal Radiology: An Overview of Novel MRI- and CT-Based Approaches. Investig. Radiol..

[18] Wang Y., Sun D., Zhang J., Kong Y., Morelli J.N., Wen D., Wu G., Li X. (2024). Multi-sequence MRI-based radiomics: An objective method to diagnose early-stage osteonecrosis of the femoral head. Eur. J. Radiol..

[19] Montin E., Kijowski R., Youm T., Lattanzi R. (2023). A radiomics approach to the diagnosis of femoroacetabular impingement. Front. Radiol..

[20] Tricco A.C., Lillie E., Zarin W., O’Brien K.K., Colquhoun H., Levac D., Moher D., Peters M.D.J., Horsley T., Weeks L. (2018). PRISMA Extension for Scoping Reviews (PRISMA-ScR): Checklist and Explanation. Ann. Intern. Med..

[21] Kim S., Kim B.R., Chae H.-D., Lee J., Ye S.-J., Kim D.H., Hong S.H., Choi J.-Y., Yoo H.J. (2022). Deep Radiomics–based Approach to the Diagnosis of Osteoporosis Using Hip Radiographs. Radiol. Artif. Intell..

[22] Yuan J., Li B., Zhang C., Wang J., Huang B., Ma L. (2025). Machine Learning-Based CT Radiomics Model to Predict the Risk of Hip Fragility Fracture. Acad. Radiol..

[23] Martel D., Monga A., Chang G. (2023). Radiomic analysis of the proximal femur in osteoporosis women using 3T MRI. Front. Radiol..

[24] Lim H.K., Ha H.I., Park S.Y., Han J. (2021). Prediction of femoral osteoporosis using machine-learning analysis with radiomics features and abdomen-pelvic CT: A retrospective single center preliminary study. PLoS ONE.

[25] Park M.S., Ha H.I., Lim H.K., Han J., Pak S. (2024). Femoral osteoporosis prediction model using autosegmentation and machine learning analysis with PyRadiomics on abdomen-pelvic computed tomography (CT). Quant. Imaging Med. Surg..

[26] Fang K., Zheng X., Lin X., Dai Z. (2024). Unveiling Osteoporosis Through Radiomics Analysis of Hip CT Imaging. Acad. Radiol..

[27] Du C., He J., Cheng Q., Hu M., Zhang J., Shen J., Wang S., Liu Y., Li J., Wei W. (2025). Automated opportunistic screening for osteoporosis using deep learning-based automatic segmentation and radiomics on proximal femur images from low-dose abdominal CT. BMC Musculoskelet. Disord..

[28] Montin E., Kijowski R., Youm T., Lattanzi R. (2024). Radiomics features outperform standard radiological measurements in detecting femoroacetabular impingement on three-dimensional magnetic resonance imaging. J. Orthop. Res..

[29] Montin E., Namireddy S., Ponniah H.S., Logishetty K., Khodarahmi I., Glyn-Jones S., Lattanzi R. (2025). Radiomics for Precision Diagnosis of FAI: How Close Are We to Clinical Translation? A Multi-Center Validation of a Single-Center Trained Model. J. Clin. Med..

[30] Montin E., Deniz C.M., Kijowski R., Youm T., Lattanzi R. (2024). The impact of data augmentation and transfer learning on the performance of deep learning models for the segmentation of the hip on 3D magnetic resonance images. Inform. Med. Unlocked.

[31] Alkhatatbeh T., Alkhatatbeh A., Guo Q., Chen J., Song J., Qin X., Wei W. (2025). Interpretable machine learning and radiomics in hip MRI diagnostics: Comparing ONFH and OA predictions to experts. Front. Immunol..

[32] Klontzas M.E., Manikis G.C., Nikiforaki K., Vassalou E.E., Spanakis K., Stathis I., Kakkos G.A., Matthaiou N., Zibis A.H., Marias K. (2021). Radiomics and Machine Learning Can Differentiate Transient Osteoporosis from Avascular Necrosis of the Hip. Diagnostics.

[33] Batur H., Rasit Mendi B.A., Cay N. (2023). Bone marrow lesions of the femoral head: Can radiomics distinguish whether it is reversible?. Pol. J. Radiol..

[34] He Y., Chen Y., Chen Y., Li P., Yuan L., Ma M., Liu Y., He W., Zhou W., Chen L. (2025). X-ray based radiomics machine learning models for predicting collapse of early-stage osteonecrosis of femoral head. Sci. Rep..

[35] Gao S., Zhu H., Wen M., He W., Wu Y., Li Z., Peng J. (2024). Prediction of femoral head collapse in osteonecrosis using deep learning segmentation and radiomics texture analysis of MRI. BMC Med. Inform. Decis. Mak..

[36] Jia D., Zhang Y., Li H., Guo C., Wu Y., Shi X., Yang L., Mo J., Liu X., Xu Y. (2024). Predicting steroid-induced osteonecrosis of the femoral head: Role of lipid metabolism biomarkers and radiomics in young and middle-aged adults. J. Orthop. Surg..

[37] He B., Zhang X., Peng S., Zeng D., Chen H., Liang Z., Zhong H., Ouyang H. (2024). Prediction of intraoperative press-fit stability of the acetabular cup in total hip arthroplasty using radiomics-based machine learning models. Eur. J. Radiol..

[38] Zheng X., Xiao C., Xie Z., Liu L., Chen Y. (2022). Prediction Models for Prognosis of Femoral Neck–Fracture Patients 6 Months after Total Hip Arthroplasty. Int. J. Gen. Med..

