# Supplementary material for: The Impact of Radiomics Image Analysis on Adult Hip Pathologies: A Scoping Review
Source: J Clin Med. 2026 Feb 9;15(4):1366. doi: 10.3390/jcm15041366 (PMC12942101; doi:10.3390/jcm15041366)
Supplement: Supplementary file 1 [file jcm-15-01366-s001.zip › jcm-4097696-supplementary.pdf]

File S1.

Full Search Strategy (Databases, Strings, Filters, and Deduplication)

Databases queried: PubMed (MEDLINE), Embase and Scopus (Elsevier).

Time window: 1 Jan 2021 – 30 Aug 2025 (chosen to capture contemporary radiomics/AI pipelines).

Last search executed: 30 Aug 2025.

Languages: English (others excluded).

Population: Adults (≥18 years). Human studies only.

Study types included (primary analysis): Original peer-reviewed research articles (including "articles in press"/Epub ahead of print).

Study types excluded (primary analysis): Reviews, editorials, letters/notes, conference abstracts/reviews, case reports/series, preclinical (animal/cadaver/phantom).

Snowballing: Backward/forward citation chasing on included records; for this phase, reviews were screened for references but not included in synthesis.

Note on filters: We applied a Humans filter. We did **not** apply strict "Adult" age filters in the databases to avoid inadvertent loss of adult-inclusive cohorts; pediatric exclusion was enforced at screening.

A. PubMed / MEDLINE

Field tags used: [tiab] for Title/Abstract; [Mesh] for MeSH.

Limits: Humans; publication date 2021/01/01–2025/08/30; exclude non-eligible publication types.

Primary strategy (radiomics-focused, plus essential AI terms):

(("Radiomics"[Mesh] OR radiomic*[tiab] OR "texture analy*"[tiab] OR "radiomic feature*"[tiab] OR "radiomic signature*"[tiab] OR radiogenom*[tiab] OR "deep learning"[tiab] OR "machine learning"[tiab] OR "artificial intelligence"[tiab] OR "convolutional neural network*"[tiab] OR neural network*[tiab] OR CNN[tiab]) AND ("Hip Joint"[Mesh] OR "Osteoarthritis, Hip"[Mesh] OR "Hip Fractures"[Mesh] OR "Femur Head Necrosis"[Mesh] OR "Arthroplasty, Replacement, Hip"[Mesh] OR hip[tiab] OR "hip joint"[tiab] OR acetabul*[tiab] OR femoroacetab*[tiab] OR FAI[tiab] OR "femoroacetabular impingement"[tiab] OR "femoral head"[tiab] OR "femoral neck"[tiab] OR "proximal femur"[tiab] OR ONFH[tiab] OR osteonecrosis[tiab] OR "avascular necrosis"[tiab] OR "hip fracture*"[tiab] OR "femoral neck fracture*"[tiab] OR "intertrochanteric fracture*"[tiab] OR "hip osteoarthritis"[tiab] OR "total hip arthroplasty"[tiab] OR THA[tiab] OR "hip replacement"[tiab]) AND ("Tomography, X-Ray Computed"[Mesh] OR "Magnetic Resonance Imaging"[Mesh] OR "Radiography"[Mesh] OR "Dual-Energy X-Ray Absorptiometry"[Mesh] OR CT[tiab] OR "computed tomography"[tiab] OR MRI[tiab] OR "magnetic resonance"[tiab] OR radiograph*[tiab] OR "x-ray*"[tiab] OR DXA[tiab] OR densitometr*[tiab])) AND ("2021/01/01"[Date - Publication] : "2025/08/30"[Date - Publication]) AND Humans[Mesh] NOT (Review[Publication Type] OR Editorial[Publication Type] OR Letter[Publication Type] OR Case Reports[Publication Type]) NOT (animals[mh] NOT humans[mh])

B. Embase (Embase.com)

Syntax: Emtree exploded terms /exp, title/abstract: ti,ab, limits/lim, publication years/py. **Limits:** Humans; years 2021–2025; exclude non-eligible types (including conference abstracts/reviews).

Primary strategy (radiomics-focused, plus essential AI terms):

('radiomics'/exp OR radiomic*:ti,ab OR 'texture analysis'/exp OR 'texture analy*':ti,ab OR radiogenom*:ti,ab OR 'machine learning'/exp OR 'deep learning'/exp OR 'convolutional neural network'/exp OR ('neural network*' OR 'convolutional neural network*' OR cnn):ti,ab AND ('hip joint'/exp OR hip:ti,ab OR acetabul*:ti,ab OR femoroacetab*:ti,ab OR fai:ti,ab OR 'femoroacetabular impingement'/exp OR 'femoral head'/exp OR 'femoral neck'/exp OR 'proximal femur':ti,ab OR onfh:ti,ab OR 'osteonecrosis'/exp OR 'avascular necrosis':ti,ab OR 'hip fracture'/exp OR 'femoral neck fracture'/exp OR 'intertrochanteric fracture'/exp OR 'hip osteoarthritis'/exp OR 'hip arthroplasty'/exp OR 'arthroplasty, replacement, hip'/exp OR 'total hip arthroplasty':ti,ab OR tha:ti,ab) AND ('computed tomography'/exp OR 'magnetic resonance imaging'/exp OR 'x ray'/exp OR 'radiography'/exp OR 'dual energy x ray absorptiometry'/exp OR ct:ti,ab OR mri:ti,ab OR radiograph*:ti,ab OR 'x-ray*':ti,ab OR dxa:ti,ab OR densitometr*:ti,ab AND [2021-2025]/py AND [humans]/lim NOT ([conference abstract]/lim OR [conference review]/lim OR [editorial]/it OR [letter]/it OR [note]/it OR [case report]/it OR [review]/it)

C. Scopus

Syntax: TITLE-ABS-KEY(), PUBYEAR, DOCTYPE.

Limits: DOCTYPE = article (includes “article in press”); PUBYEAR 2021–2025; English or Italian.

Primary strategy (radiomics-focused, plus essential AI terms):

(TITLE-ABS-KEY(radiomic* OR "texture analy*" OR "radiomic feature*" OR "radiomic signature*" OR radiogenom* OR "machine learning" OR "deep learning" OR "convolutional neural network*" OR cnn) AND TITLE-ABS-KEY(hip OR "hip joint" OR acetabul* OR femoroacetab* OR FAI OR "femoroacetabular impingement" OR "femoral head" OR "femoral neck" OR "proximal femur" OR ONFH OR osteonecrosis OR "avascular necrosis" OR "hip fracture*" OR "femoral neck fracture*" OR "intertrochanteric fracture*" OR "hip osteoarthritis" OR "total hip arthroplasty" OR THA OR "hip replacement") AND TITLE-ABS-KEY(CT OR "computed tomography" OR MRI OR "magnetic resonance" OR radiograph* OR "x-ray*" OR DXA OR densitometr*)) AND (PUBYEAR > 2020 AND PUBYEAR < 2026) AND DOCTYPE(ar)
